# Low Phase Noise, Dual-Frequency Pierce MEMS Oscillators with Direct Print Additively Manufactured Amplifier Circuits

**DOI:** 10.3390/mi16070755

**Published:** 2025-06-26

**Authors:** Liguan Li, Di Lan, Xu Han, Tinghung Liu, Julio Dewdney, Adnan Zaman, Ugur Guneroglu, Carlos Molina Martinez, Jing Wang

**Affiliations:** 1 Department of Electrical Engineering, University of South Florida, 4202 E. Fowler Avenue, Tampa, FL 33620, USA; ugur@usf.edu (U.G.); carlosm2@usf.edu (C.M.M.); 2 Coherence. Inc., 375 Saxonburg Boulevard, Saxonburg, PA 16056, USA; di.lan@coherent.com; 3 Qorvo, Inc., Greensboro, NC 27401, USA; xu.han@qorvo.com; 4 Skyworks Solutions, Inc., 5260 California Avenue, Irvine, CA 92617, USA; tinghungliu1123@gmail.com (T.L.); julio.dewdneymontero@skyworksinc.com (J.D.); 5 Microelectronics and Semiconductor Institute, King Abdulaziz City for Science and Technology (KACST), Riyadh 11442, Saudi Arabia; azaman@kacst.edu.sa

**Keywords:** additive manufacturing, advanced packaging, phase noise, piezoelectric, zinc oxide (ZnO), silicon-on-insulator (SOI), MEMS, resonators, oscillator, quality factor

## Abstract

This paper presents the first demonstration and comparison of two identical oscillator circuits employing piezoelectric zinc oxide (ZnO) microelectromechanical systems (MEMS) resonators, implemented on conventional printed-circuit-board (PCB) and three-dimensional (3D)-printed acrylonitrile butadiene styrene (ABS) substrates. Both oscillators operate simultaneously at dual frequencies (260 MHz and 437 MHz) without the need for additional circuitry. The MEMS resonators, fabricated on silicon-on-insulator (SOI) wafers, exhibit high-quality factors (*Q*), ensuring superior phase noise performance. Experimental results indicate that the oscillator packaged using 3D-printed chip-carrier assembly achieves a 2–3 dB improvement in phase noise compared to the PCB-based oscillator, attributed to the ABS substrate’s lower dielectric loss and reduced parasitic effects at radio frequency (RF). Specifically, phase noise values between −84 and −77 dBc/Hz at 1 kHz offset and a noise floor of −163 dBc/Hz at far-from-carrier offset were achieved. Additionally, the 3D-printed ABS-based oscillator delivers notably higher output power (4.575 dBm at 260 MHz and 0.147 dBm at 437 MHz). To facilitate modular characterization, advanced packaging techniques leveraging precise 3D-printed encapsulation with sub-100 μm lateral interconnects were employed. These ensured robust packaging integrity without compromising oscillator performance. Furthermore, a comparison between two transistor technologies—a silicon germanium (SiGe) heterojunction bipolar transistor (HBT) and an enhancement-mode pseudomorphic high-electron-mobility transistor (E-pHEMT)—demonstrated that SiGe HBT transistors provide superior phase noise characteristics at close-to-carrier offset frequencies, with a significant 11 dB improvement observed at 1 kHz offset. These results highlight the promising potential of 3D-printed chip-carrier packaging techniques in high-performance MEMS oscillator applications.

## 1. Introduction

Timing accuracy and stability are essential for the reliable performance of radio frequency (RF) transceiver systems and other electronic applications requiring precise frequency references for signal mixing and modulation [1]. Oscillators address this need by generating stable output signals at desired frequencies without external input. Typically, oscillators comprise a resonator and a transistor-based sustaining amplifier arranged in a closed-loop configuration, where oscillation occurs once the transfer function yields complex conjugate poles positioned precisely on the imaginary axis. The primary performance metrics of oscillators—phase noise, frequency stability, and temperature sensitivity—are predominantly governed by the properties of the resonators and the packaging assembly.

Quartz oscillators have historically dominated this field due to their exceptional stability, low phase noise, and consistent reliability across the operating temperature range. Quartz resonators exhibit inherently high-quality factors (*Q*), facilitating superior oscillator performance [2,3]. However, quartz technology faces limitations, particularly in the ultra-high-frequency (UHF) range and beyond, as increased frequency synthesizer up-conversion ratios can degrade system performance with respect to phase noise significantly [4]. Thus, microelectromechanical systems (MEMS) technology has emerged as a compelling alternative, overcoming the aforementioned constraints associated with quartz-based resonators. MEMS-based oscillators can achieve comparable or superior performance metrics at substantially higher frequencies—up to several GHz—and can be mass-produced with significantly reduced form factors (one or two magnitudes smaller than quartz crystals). Also, MEMS resonators are inherently compatible with integrated circuit (IC) foundry processes, enabling greater integration density and substantial reductions in manufacturing costs. Thus, MEMS oscillators represent a crucial advancement for frequency reference technologies, addressing modern electronic systems’ evolving demands [2,5,6,7].

MEMS resonators [8] are primarily classified according to their transduction mechanisms—capacitive [1,9,10,11,12] and piezoelectric [10,13,14,15]. Capacitive MEMS resonators have demonstrated outstanding performance in ultra-low phase noise and/or low-power oscillators, achieving phase noise as low as −110 dBc/Hz at a 1 kHz offset, attributable to their exceptionally high-quality factors exceeding 48,000 [16]. These resonators deliver precise frequency control and impressive figure of merit (FOM), rivaling quartz counterparts [17]. Nevertheless, capacitively transduced MEMS resonators inherently suffer from high motional impedance (often exceeding 10 kΩ) [18,19], limiting output power and necessitating complex circuitry, including high-voltage charge pumps to generate the needed direct-current (DC) bias voltage range of 32.6 V [20] and specialized oscillator architectures. Consequently, the integration complexity and power consumption increase significantly.

In contrast, piezoelectrically transduced MEMS resonators, such as those based on thin-film ZnO or aluminum nitride (AlN), provide a lower motional impedance while maintaining adequate *Q* (e.g., a motional impedance of 350 Ω with a quality factor of 172, 105) [21], thereby enabling simpler oscillator architectures with improved signal strength and lower power requirements. Piezoelectric MEMS resonator technology has garnered significant interests for both timing and filtering applications, including surface-acoustic-wave (SAW) [22,23] and bulk-acoustic-wave (BAW) devices [23,24,25]. A DC bias voltage is not needed as compared to the capacitive counterparts, thus leading to a much simpler circuit. Furthermore, piezoelectric transduction allows for the generation of multiple frequency outputs from a single resonator operating in different modes, enhancing design flexibility for RF systems.

Despite advances in MEMS resonator design, packaging remains a critical bottleneck influencing oscillator performance, reliability, and manufacturability. Integrating MEMS devices with RF circuitry typically requires wire bonding, which introduces parasitic effects, limits scalability, and requires performance-demanding printed-circuit-board (PCB) implementation. To overcome these issues, we explore an innovative packaging strategy using 3D-printed interconnects and encapsulation with acrylonitrile butadiene styrene (ABS). By implementing oscillator circuits on both conventional printed-circuit-boards (PCBs) and 3D-printed ABS chip-carrier substrates, we can assess the impact of printed substrate material and packaging configuration on key performance metrics such as phase noise and output power. This hybrid system-in-package (SiP) approach enables modular testing and offers new possibilities for rapid prototyping and system-level integration of MEMS-based oscillators and frequency synthesizers. The Pierce oscillator design, widely adopted for frequency generation, is typically composed of a resonator and an active sustaining amplifier arranged in a closed-loop configuration [26,27]. The specific implementation of this topology depends on the selected transistor’s characteristics. In MEMS-based oscillators, the desire for monolithic integration often necessitates wire bonding between the resonator and amplifier, a process that introduces variability and detrimental parasitic effects and limits yield. For instance, researchers have designed and fabricated oscillator circuits by a complementary metal-oxide-semiconductor (CMOS) foundry, followed by wire bonding to MEMS resonators [28,29].

There is a great demand for advanced packaging technology in place of printed-circuit-board and wire bonding technology, which plays a pivotal role in the heterogeneous integration of RF electronics, such as the implementation of MEMS oscillators. It is anticipated for such advanced packaging technology to enable IC chip encapsulation, allow the chips to achieve higher levels of integration and performance, and offer higher packaging density [30,31,32]. Advanced packaging can provide the convenience of testing modular components or circuit behavior, such as the ability to measure the open-loop gain of the oscillator circuit and then complete the packaging assembly into a closed-loop oscillator circuit through interconnect technology. The packaging with 3D-printed ABS material [31,33,34,35,36] along with sub-hundred micrometer, low-parasitic lateral interconnects are implemented in the form of chip-carrier assembly and sealed with a dome-shaped ABS encapsulation without negatively impacting performance. The benefits of a 3D-printed packaging assembly enable us to customize the sustaining amplifier design after characterization of the MEMS resonator-based oscillator, so that one of the resonances can be chosen to act as the tank circuit. The convenience of a tailor-designed system-in-package oscillator by 3D-printing is crucial to reduce the design-to-fabrication cycle, while not sacrificing phase noise performance.

Recently, 3D additive manufacturing (AM) has gained considerable interest from the research community due to its ability to produce high quality RF circuits comparable to traditional printed-circuit-board (PCB) technology [32]. Nevertheless, performance enhancements remain necessary due to the inherent limitations of lossy dielectric materials suitable for 3D-printing. In this work, a MEMS-based Pierce oscillator circuit, fabricated using fused deposition modeling (FDM) and microdispensing technologies [37,38,39], is introduced. FDM and ABS substrate (ε_r_ = 2.1 and tan *δ* = 0.0058 at 17 GHz) were selected for their ease of fabrication, cost-effectiveness, and compatibility with 3D-printing [34,39,40,41]. Advanced packaging strategies utilizing 3D-printed ABS chip-carriers with encapsulation and lateral interconnects provide high integration density and enhanced device performance, facilitating the rapid prototyping of Pierce oscillator designs and substantially reducing the design-to-fabrication cycle without compromising phase noise performance.

Our prior work and preliminary results of dual-frequency MEMS-based oscillators using a single ZnO-on-SOI resonator and 3D-printed low-loss lateral interconnects have been reported in [42,43], respectively. In this paper, the background related to the MEMS resonator and Pierce oscillator is detailed in Section 2, including a short discussion of the phase noise and 3D-printing process. The experimental results of dual-frequency MEMS-based oscillators are presented in Section 3, including the comparison of the measured phase noise results of PCB HBT oscillator, PCB E-pHEMT oscillator, and 3D-printed HBT oscillator. All the measurements are based on a 250 µm × 91 µm rectangular plate piezoelectric ZnO-on-SOI MEMS resonator, featuring 13 equally spaced IDT fingers as the top electrode and 13 side-supporting anchors. Three identically designed and concurrently fabricated MEMS resonators have been used for three types of packaging implementation of Pierce oscillators, including PCB HBT oscillator, PCB E-pHEMT oscillator, and HBT oscillator on 3D-printed ABS chip-carriers. Accordingly, the findings reported herein should be viewed as preliminary. Future research will aim to improve fabrication tolerance, repeatability, and reliability, with a particular focus on leveraging additive manufacturing for advanced circuit integration, structural electronics packaging, and heterogeneous system integration.

## 2. Materials and Methods

### 2.1. Phase Noise

In a transceiver block diagram, phase-locked loop (PLL) is one of the most important modules used for synthesizing frequency reference signals by simultaneously relating output signals to the phase of the input signals. From a classical integer-*N* PLL block diagram, the output frequency is *N* times of the frequency of the reference oscillator; however, such frequency multiplication would generally result in an increase in phase noise by 20 × log(*N*) as a direct detrimental effect. Therefore, it is crucial to retain the lowest achievable phase noise by the oscillator design and implementation, which in turn mitigates the distortion of the desired signal and negative impact on the performance of the entire system [44,45]. Equation (1) presents a classical Leeson’s phase noise model, which is the most widely used linear model for phase noise. The equation for the phase noise model of an oscillator is given by [44]:

(1) $$L\left(∆\omega \right)=10log\left[\frac{2FkT}{P_{sig}}\cdot \left(1+{\left(\frac{\omega_{0}}{2Q∆\omega }\right)}^{2}\right)\cdot \left(1+\frac{{∆\omega }_{1/f^{3}}}{\left|∆\omega \right|}\right)\right]\;$$

where *F* is the effective noise figure of the sustaining amplifier, k is the Boltzmann constant, *T* is the operating temperature, *P*sig is the output power of the oscillator, ω_0_ is the oscillation frequency of the oscillator, ∆ω is the offset frequency at which the phase noise is measured, *Q* is the loaded quality factor of the resonator, and ∆ω_1/f_^3^ is the corner offset frequency, which is mostly determined by the amplifier’ nonlinearity. Leeson’s model is an empirical model based on the measured noise figure and corner offset frequency. In a typical scenario, the phase noise starts to decrease at a rate of −30 dB per decade until it reaches the corner offset frequency. Then, within the half-bandwidth of the resonator (i.e., ω_0_/2*Q*), the phase noise decreases at −20 dB per decade. The far-away noise floor depends on the noise figure and the oscillator’s output amplitude, as seen in Equation (1). Obviously, another important factor in achieving a lower phase noise is to adopt a high-*Q* resonator as the tank circuit, which is realized in the present work through MEMS resonators.

### 2.2. Piezoelectric ZnO-on-SOI MEMS Resonators

As previously discussed, the performance of the selected resonator plays a pivotal role in determining the phase noise of the designed MEMS oscillator. One of the figure of merits (FOM) is generally defined as the product of the resonance frequency and the quality factor (*f*_0_ × *Q*). Quartz resonator can provide extremely high-*Q*, while the highest operation frequency is limited to very-high-frequency (VHF) range [46,47,48]. To address this issue, piezoelectric MEMS resonators are considered in this work to generate resonance outputs at dual frequencies without complicating the sustaining amplifier circuits while providing sufficiently high-*Q* to ensure a low phase noise. Besides considering the FOM constraints, oscillator designers also need to make sure the resonator has acceptable motional resistance (or low insertion loss), so that the oscillation condition can be met. A resonator with a higher insertion loss requires an amplifier with a higher gain to compensate for the higher loss, under which conditions the low phase noise could be degraded.

**Figure 1:**
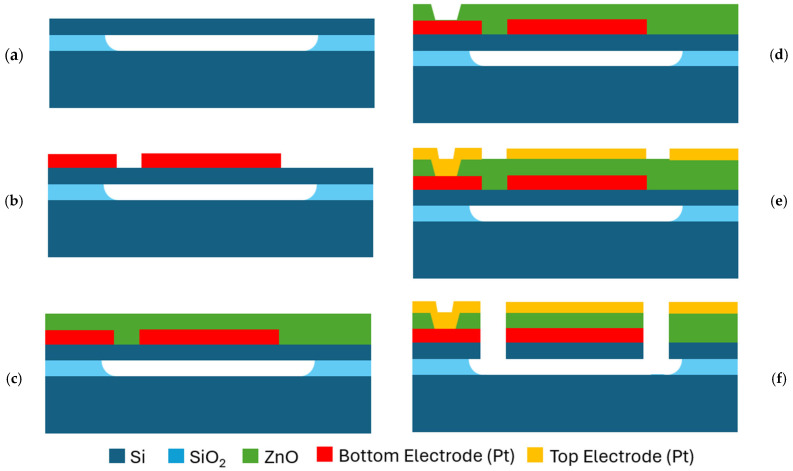
Step-by-step fabrication process of piezoelectric ZnO-on-SOI MEMS resonators: (**a**) resonator pre-release; (**b**) bottom electrode deposition and patterned by lift-off; (**c**) sputtering deposition of the piezoelectric ZnO film; (**d**) etch vias through ZnO for the bottom electrodes access; (**e**) deposition and patterning of top electrodes; and (**f**) ZnO dry etch followed by silicon dry etch.

In this work, lateral-extensional mode piezoelectric ZnO-on-SOI MEMS resonators have been fabricated through the processes as shown in Figure 1. The piezoelectric resonators have been fabricated using a silicon-on-insulator (SOI) wafer and a five-mask photolithography process. A SOI wafer with a 2 μm-thick buried oxide (BOX) layer and a 10 μm-thick single-crystal silicon device layer are employed, which leads to the optimal trade-off between the best-achievable motional resistance and the quality factor [49]. As silicon device layer gets thinner, the ZnO piezoelectric transducer layer becomes a larger portion of the resonator body, therefore increasing the electromechanical coupling coefficient. However, at the same time, a slight degradation in the quality factor (*Q)* is expected due to a larger acoustic loss contributions in the ZnO layer as compared to that of single- crystal silicon of a SOI wafer.

The fabrication process began with a pre-release process in which a series of released holes were etched around the resonator body in the silicon device layer, followed by wet isotropic etching to remove the buried oxide layer underneath using a 49% hydrofluoric acid (HF) solution, as shown in Figure 1a. The bottom electrodes were formed by sputtering a 30 nm-thick chrome (Cr) adhesion layer and 170 nm-thick platinum (Pt) as the main conductor layer. Then, the bottom electrodes were patterned utilizing a lift-off process, as shown in Figure 1b, followed by sputtering deposition of a 500 nm-thick ZnO piezoelectric layer, as depicted in Figure 1c. Subsequently, openings to contact the bottom electrodes through ZnO were wet-etched in a mixed solution of hydrochloric acid (HCl)/water (H_2_O) with a ratio of 1/200, as shown in Figure 1d. Furthermore, 200 nm-thick platinum (Pt) top electrodes were then deposited and patterned by lift-off, as illustrated in Figure 1e. The ZnO was dry etched using a mixture of methane (CH_4_) and argon (Ar) gases (CH_4_-Ar) plasma via reactive ion etching. Finally, the device resonator body was defined and released by a silicon anisotropic deep reactive ion etching (DRIE) process to cut through the pre-released device layer already suspended over the SOI substrate, as shown in Figure 1f.

Piezoelectrically-transduced resonators not only eliminate the need for a DC biasing for their operations but also exhibit relatively low motional impedances, often lower than 1 kΩ, which positions them as an excellent candidate for reference oscillators [44,50]. The basic transduction mechanism for a piezoelectric lateral-extensional mode resonator exploits a pair of interdigitated transducer (IDT) electrodes [44,51,52] to excite the targeted width-extensional mode. When the IDT top electrodes, piezoelectric layer, and bottom ground electrode are strategically designed, multi-resonance frequencies can be excited in a single resonator when the piezoelectric transducer layer sandwiched by the top and bottom electrodes undergoes modal vibration to trigger multiple resonance modes.

**Figure 2:**
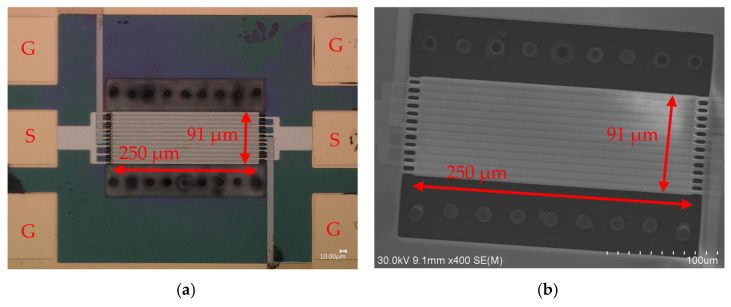
The top-view photos of a 250 µm × 91 µm ZnO-on-SOI rectangular plate resonator along with its key dimensions, including (**a**) Keyence VHX-6000 digital microscope image, where G and S represent the ground and signal probe pads, respectively; and (**b**) SEM photo of this resonator device.

A 250 µm × 91 µm rectangular plate piezoelectric ZnO-on-SOI MEMS resonator with 13 equally spaced IDT fingers as the top electrode and 13 side-supporting anchors is investigated in this work, which corresponds to an IDT pitch size of slightly less than 7 µm due to the finite IDT finger width. Figure 2 presents the top-view microscope and scanning-electron-microscope (SEM) images of a 250 µm × 91 µm rectangular plate resonator microfabricated using the processes depicted in Figure 1. The resonator is designed to operate at a 13th-order width-extensional mode, which was characterized to exhibit two distinct width-extensional modes at 259.7 MHz and 436.4 MHz, respectively. The 13 side-supporting anchors located at the nodal locations of the target mode shape suppress the spurious modes through anchor-related dissipations. The resonance frequency of an nth-order symmetrical width lateral-extensional mode resonator is given by [49,51,52,53], thus:

(2) $$v_{eq}=\sqrt{\frac{E_{dev}T_{dev}+E_{be}T_{be}+E_{p}T_{p}+E_{te}T_{te}}{\left(\rho_{dev}T_{dev}+\rho_{be}T_{be}+\rho_{p}T_{p}+\rho_{te}T_{te})(1-\sigma^{2}\right)}}\;$$

(3) $$f_{n}=\frac{n}{2W}v_{eq}\;$$

where $v_{eq}$ is the equivalent acoustic velocity for stacked layers, while *E* is the Young’s modulus, *T* is the thickness, *ρ* is the density, and σ is the Poisson’s ratio. The subscripts dev, be, p, and te refer to the device layer, bottom electrode, piezoelectric layer, and top electrode, respectively. $f_{n}$ is the resonance frequency, *n* is the order of the mode, and *W* is the width of the rectangular plate resonator body.

Ground-signal-ground (GSG) probe pads for input and output terminals were designed with ground pads surrounding the MEMS resonator body to shield it. The input and output terminals are split into a total of 13 interdigitated top electrode fingers situated on the top of the ZnO piezoelectric layer to capture the piezoelectrically transduced strain field signal. Figure 2 shows two rows of pre-release holes aligned in parallel to the resonator body. The rectangular plate resonator body is suspended above the air cavity, as the buried oxide (BOX) layer was previously wet-etched through these pre-release holes.

### 2.3. Customized Pierce Oscillator Circuit for Selected Transistors

**Figure 3:**
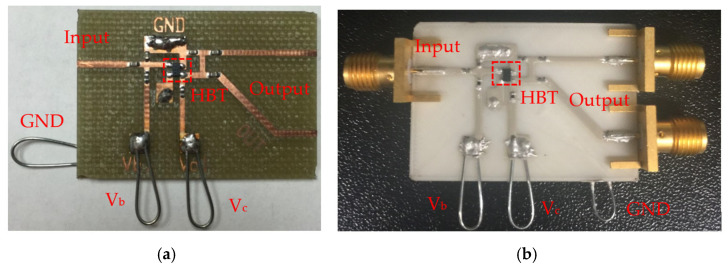
(**a**) A MEMS-based common-emitter oscillator circuit designed and milled on a PCB board, and (**b**) a 3D-printed chip-carrier assembly for a MEMS oscillator using ABS as the substrate material.

In this work, we propose an innovative approach by constructing the Pierce oscillator using discrete transmission lines and lumped components on both PCBs, as seen in Figure 3a, and a 3D-printed ABS chip-carrier substrate, as seen in Figure 3b. To our knowledge, this is the first demonstration of a Pierce oscillator topology realized through 3D-printed chip-carrier assembly with transmission lines and interconnects. Furthermore, we also introduce a modular configuration wherein coaxial cables connect the oscillator loop to multiple MEMS resonators, allowing flexible testing of devices with resonance frequencies within the amplifier’s operational bandwidth. The oscillator circuits are modeled using Keysight’s Advanced Design System (ADS) simulation, and measurements closely match simulated results. Our oscillator system integrates custom-fabricated thin-film ZnO piezoelectric MEMS resonators and commercially available SiGe HBT (California Eastern Laboratories (CEL), USA) and E-pHEMT (Avago Technologies, Mansfield, TX, USA) transistors. ZnO instead of AlN was chosen as the piezoelectric transducer material due to deposition tool limitations in our facility, while the selected transistors were chosen for their low flicker noise characteristics. The implemented oscillators demonstrate competitive phase noise performance on par with prior MEMS-based implementations, validating the effectiveness of the 3D-printed packaging and modular design approach.

**Figure 4:**
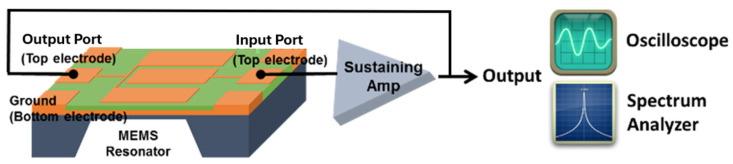
A conceptual illustration of a MEMS resonator-based oscillator circuit diagram, where green and orange color areas represent the piezoelectric ZnO film and the IDT electrode scheme of the piezoelectrically-transduced MEMS resonator, respectively.

As shown in Figure 4, when the sustaining amplifier is switched on, the electronic noise (mostly the flicker noise, also known as 1/f noise) in the circuit consistently supplies a non-zero signal to initiate and maintain the oscillation condition. The noise travels around the loop and is amplified and filtered until the oscillator output swiftly converges on a sinusoidal wave at a single frequency determined by the MEMS resonator [44].

The two-port open-loop analysis method is preferred for piezoelectric resonator-based oscillator design. Not only it can streamline the design procedure by simulating the open-loop gain and phase responses, but also the necessary starting conditions of the oscillator can be determined from the open-loop Bode response. The oscillation condition is defined by Heinrich Barkhausen, and it is given in Equations (4) and (5) for the loop gain and the loop phase conditions, respectively [44]:

(4) $$\left|A\left(s\right)\beta \left(s\right)\right|>1$$

(5) $$∠\left(A(s)\beta (s)\right)=n360^{{}^{\circ}},n=0,1,2\ldots$$

For crystal oscillators, there are three widely adopted electronic circuit schemes, including Pierce, Colpitts, and Hartley oscillator circuits, which differ based on the positive feedback design for sustaining the oscillation. In this work, we have adopted the classic Pierce circuit topology, for which a common-source Field Effect Transistor (FET) amplifier or a common-emitter Bipolar Junction Transistor (BJT) amplifier has been designed as the sustaining amplifier to provide excellent stability and reliability. Nevertheless, one single dual-mode resonator, in theory, can lead to different oscillation frequencies, as long as the aforementioned Heinrich Barkhausen oscillation conditions can be fulfilled. By precisely controlling the transistor’s biasing current, the oscillation criterion is only strategically satisfied for one chosen output frequency at a time. The open-loop gain condition for this Pierce oscillator that is predominantly dependent on the transconductance of the FET or BJT is particularly given by Equation (6), where *R*_S_ represents the equivalent motional resistance of the resonator and *g*_m_ is the transistor’s transconductance [42,44].

(6) $$\frac{g_{m}}{\omega_{0}R_{s}C_{1}C_{2}}>1\;$$

Thus, by providing a different biasing current to the chosen FET/BJT transistor that directly adjusts the transconductance gain of the sustaining amplifier (*g_m_*), the output oscillation frequency can be selected among the dual-mode frequencies of the single MEMS resonator tank circuit by properly fulfilling the loop gain criteria at the target resonance mode frequency. However, given that the gain of the sustaining amplifier typically has a high-frequency cutoff behavior, it would be necessary to increase the bias current level to ensure such a loop gain requirement can be fulfilled for the higher-frequency resonance at 437 MHz. Moreover, this resonance at 437 MHz happens to have slightly higher insertion loss as compared to that of the 260 MHz resonance (i.e., IL = 9.2 dB at 260 MHz and IL = 11.1 dB at 437 MHz). This higher insertion loss demands a higher sustaining amplifier gain by increasing the bias current.

### 2.4. 3D-Printing Process

**Figure 5:**
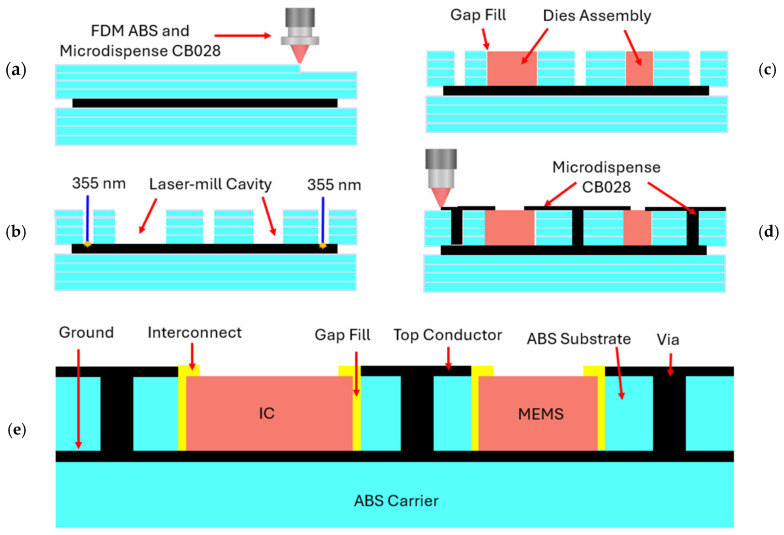
A conceptual illustration of the 3D-printing process used to implement a MEMS resonator-based Pierce oscillator in the form of system-in-package, including key steps: (**a**) the 3D-printing of the ABS substrate layer by fused deposition modeling (FDM), followed by microdispensing of CB028 ink (a silver paste)-based RF ground plane that is then covered by a FDM 3D-printed ABS layer with the thickness matching that of the MEMS and IC chips; (**b**) the laser milling of the cavity for embedding the MEMS/IC chips and needed via holes; (**c**) the insertion of the MEMS and IC dies into the designated cavities by the pick-and-place function; (**d**) the microdispensing interconnects and via hole refilling with the CB028 ink; and (**e**) the final structure of the assembled MEMS resonator-based Pierce oscillator.

The 3D-printing process used to implement a MEMS resonator-based Pierce oscillator in the form of a system-in-package is shown in Figure 5. During the via-hole laser-milling step, cavities of the same dimensions as the MEMS and IC dies are laser-milled into the 3D-printed ABS substrate to expose the CB028 bottom ground plane, as shown in Figure 5a,b. The substrate thickness is selected to match the thickness of the MEMS and IC chips. A non-conductive epoxy is used to attach the MEMS/IC chips and off-chip capacitors to the exposed CB028 ground plane to seal the dies to refill the surrounding air gaps to mitigate any potential short-circuit issues, as shown in Figure 5c. The epoxy is in liquid form when it is applied, and it is dried and solidified on the heated printer bed. Because of the low viscosity of the epoxy, it can conform within the gap between the IC die and the gap surrounding it to form a flat surface. Then, the via refilling, DC, and RF transmission lines are microdispensed using a 75 μm nozzle, while the interconnects between the MEMS and off-chip capacitors to the microstrip transmission line are micro-dispensed using a 50 μm nozzle as seen in Figure 5d. Finally, 100 µm-wide interconnects between the RF probe pads on the IC chip and the surrounding microstrip transmission lines are subsequently printed, as shown in Figure 5e.

## 3. Results and Discussion

### 3.1. Piezoelectric ZnO-on-SOI MEMS Resonators

**Figure 6:**
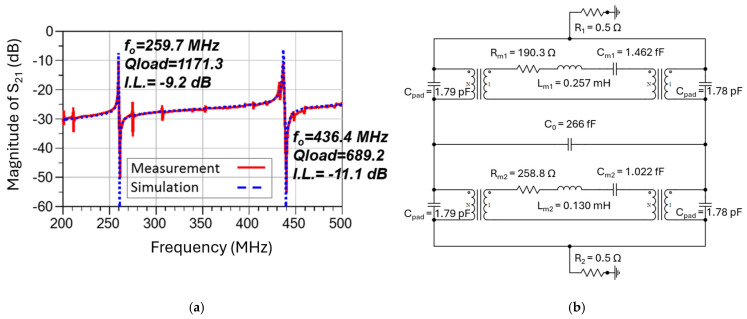
(**a**) A comparison between measured frequency responses (S_21_ in dB) and simulated results based on a full dual-mode equivalent circuit model of the ZnO-on-SOI resonator at 259.7 MHz and 436.4 MHz; and (**b**) the implemented electrical equivalent circuit model for dual-mode resonances of the ZnO-on-SOI resonator, which is employed to obtain a simulated frequency response (S_21_ in dB).

As illustrated in Figure 6a, the measured frequency response of the dual-mode resonator features the first resonance at 259.7 MHz that exhibits a loaded-*Q* of 1171.3 and an insertion loss of 9.2 dB, and the second resonance at 436.4 MHz with a loaded-*Q* of 689.2 and an insertion loss of 11.1 dB. The complete equivalent circuit model for this dual-mode MEMS resonator is shown in Figure 6b, which consists of two pairs of series inductor-capacitor-resistor (LCR) components sharing the same transducer capacitor (C_0_), representing the motional current behaviors of two different resonant modes vibrating on a single device at the frequencies of 259.7 MHz and 436.4 MHz. Two transformers are located at each side of the series LCR to simulate the piezoelectric effect, in which the energy converts back and forth between electrical and mechanical domains [42,44]. This equivalent circuit model can successfully match the measured frequency response of the resonator with the exception of the spurious modes, which can be ignored when designing a Pierce oscillator.

### 3.2. Pierce Oscillator with Selected Transistors

**Figure 7:**
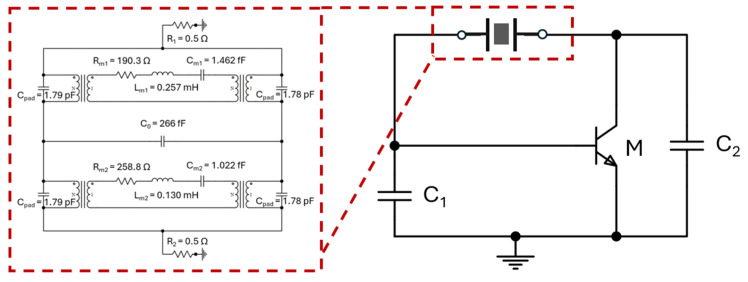
A complete Pierce oscillator circuit diagram with a single transistor (a BJT for this example) in a closed-loop configuration with two capacitors (C_1_ and C_2_) and a MEMS resonator tank circuit. During circuit simulation, an electrical equivalent circuit model for the dual-mode MEMS resonator with specifically chosen resonance frequencies was employed.

To demonstrate dual-output frequencies from this Pierce oscillator for a comparison study, a SiGe heterojunction bipolar transistor (HBT) acquired from CEL and an enhancement-mode pseudomorphic high-electron-mobility transistor (E-pHEMT) from Avago Technologies have been chosen to implement the Pierce oscillator configuration with FET and BJT transistors. Both HBT and E-pHEMT transistors have low flicker noise but different corner frequencies. The measured results indicate that the oscillator designed with a SiGe HBT transistor has shown an 11 dB- and 5 dB-lower phase noise values at 1 kHz offset as compared to that of the E-pHEMT counterpart when operating at 260 MHz and 437 MHz, respectively. As seen in Figure 7, the Pierce oscillator circuit simulation has been performed using Keysight’s Advanced Design System (ADS) (Santa Rosa, CA, USA) circuit simulator and transistor model library from Modelithics Inc. (Tampa, FL, USA) to represent the real-world behavior model of the lumped components and transistors. To assess the performance impacts of the dual-mode MEMS resonator to the rest of the closed-loop oscillator circuit, two different approaches have been used. The first approach is to directly insert the measured scattering-parameter (SP) frequency responses of the MEMS resonator as the tank circuit, and the other way is to adopt the equivalent circuit model of the dual-mode resonator, as seen Figure 6b.

**Figure 8:**
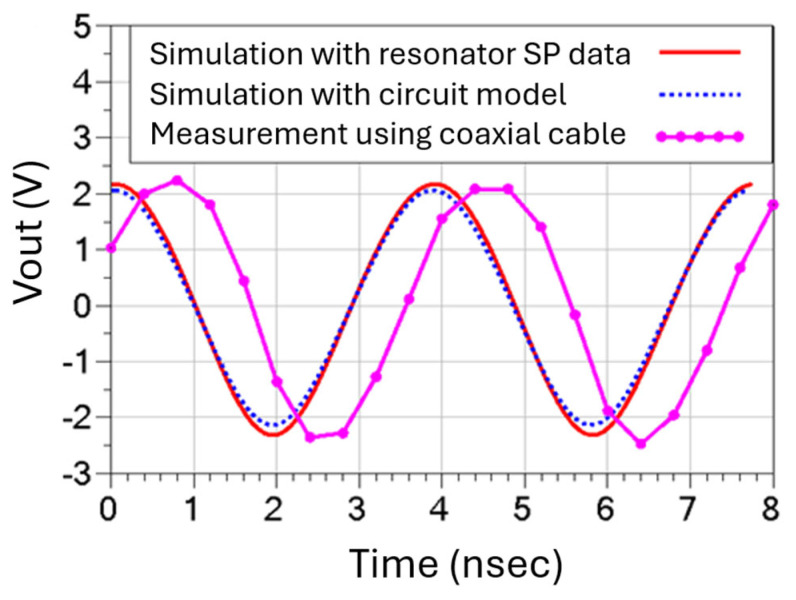
A comparison between measured time-domain waveforms generated by a MEMS oscillator operating at 260 MHz versus two types of simulated time-domain oscillator output waveforms.

For the purpose of successfully generating two stable oscillation output signals, it is also crucial to evaluate the discrepancies between the measured and simulated oscillator output waveforms. It is worthwhile mentioning that the full time-domain oscillator simulation included all the behavior models for the lumped components, such as the transistor under its chosen bias conditions, and the substrate property was suitably modified to represent the 3D-printed ABS material. Two different oscillator design approaches were adopted to integrate the resonator tank circuit with the rest of the Pierce oscillator circuit components. As shown in Figure 8, the direct “plug-in” insertion of the resonator’s scattering-parameter (SP) data in simulation works equally as well as the experimentally validated equivalent circuit model of the resonator, as shown in Figure 6.

When the connected HBT transistor is biased with 10.1 mA, the overall open-loop transfer function has an 8 dB gain margin to ensure the oscillation can be detectable after the loop is closed. As shown in Figure 8, the constructed oscillator is capable of locking into one of the designed modal frequencies of the high-*Q* MEMS resonator at 260 MHz, thus producing a stable sinusoidal output waveform. Most importantly, both the frequency and the amplitude of the measured and simulated time-domain waveforms of the output signal of the designed Pierce oscillator match well, as can be seen in Figure 8. It is observed that both peak-to-peak oscillation amplitude and the DC offset voltage are also in close agreement between measured and simulated results, including the simulation by both the equivalent circuit model and the measured scattering-parameter characteristics of the MEMS resonator.

**Figure 9:**
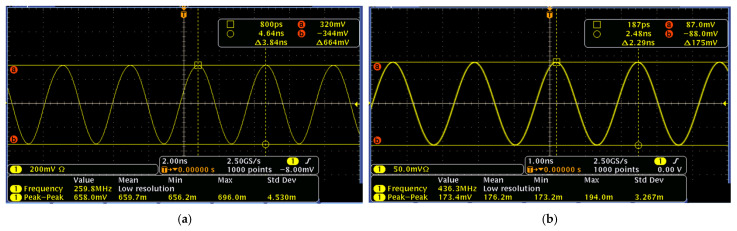
HBT transistor-based dual-mode Pierce oscillator’s output waveforms measured by an oscilloscope: (**a**) at 260 MHz with a peak-to-peak voltage of 0.664 V and (**b**) at 437 MHz with a peak-to-peak voltage of 0.175 V.

**Figure 10:**
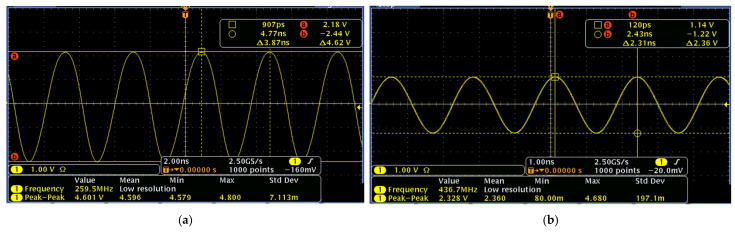
E-pHEMT transistor-based dual-mode Pierce oscillator’s output waveforms measured by an oscilloscope: (**a**) at 260 MHz with a peak-to-peak voltage of 4.62 V and (**b**) at 437 MHz with a peak-to-peak voltage of 2.36 V.

Figure 9 presents the measured dual-mode output waveforms of a Pierce oscillator, equipped with an HBT transistor, at 260 MHz with a peak-to-peak voltage of 0.664 V and 437 MHz with a peak-to-peak voltage of 0.175 V, respectively. Meanwhile, Figure 10 presents the measured output waveforms of another Pierce oscillator, designed and implemented with an E-pHEMT transistor, at 260 MHz with a peak-to-peak voltage of 4.62 V and 437 MHz with a peak-to-peak voltage of 2.36 V, respectively. It is noted that both versions of the Pierce oscillator circuits were designed to operate with the same MEMS resonator tank circuit for the purpose of directly comparing their performance. In this work, HBT transistors are strategically chosen due to their inherent low flicker noise corner frequency, which together with the high-quality factor of the MEMS resonator tank circuit, benefits the close-to-carrier phase noise. Meanwhile, the far-from-carrier phase noise often correlates closely with the peak-to-peak oscillation amplitude. Therefore, the noticeably different output waveform amplitudes in terms of peak-to-peak voltages between these two designs of Pierce oscillator implemented with HBT and E-pHEMT transistor circuits also provide us with a unique opportunity to investigate and compare these two oscillator designs in terms of both close-to-carrier and far-from-carrier phase noise characteristics.

**Figure 11:**
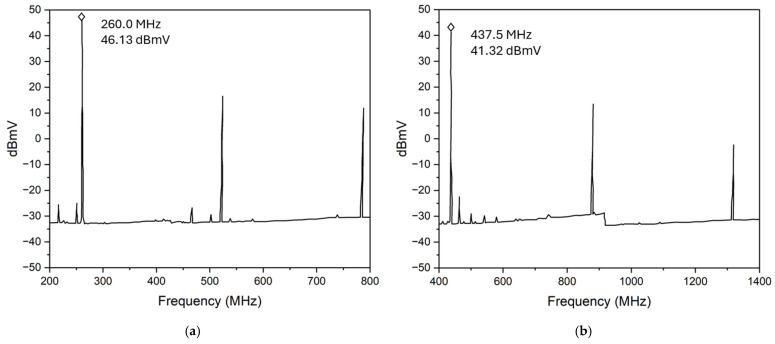
Measured broadband frequency responses of the MEMS oscillator showing two output signals: (**a**) at 260 MHz with a signal voltage of 46.13 dBmV and (**b**) at 437 MHz with a signal voltage of 41.32 dBmV, which are measured by using a spectrum analyzer.

The multi-frequency oscillation outputs are achieved by simply adjusting the biasing current of the sustaining amplifier. Under a higher transimpedance gain by increasing the bias current to 23.8 mA, the oscillator is reconfigured to oscillate at a high-band frequency of 437 MHz, which is determined by the high-frequency resonance mode. As seen in Figure 11b, the fundamental oscillation frequency output signal at 437 MHz is detected by the spectrum analyzer, and its higher-order harmonics at 874 MHz and 1.31 GHz can be easily observed. Moreover, under this particular bias condition, the higher-order harmonics of the first oscillation mode at 260 MHz of the MEMS resonator are not detected, as shown in Figure 11a, suggesting that the oscillator output has successfully locked to the resonance frequency of 437 MHz of the high-frequency mode of the dual-mode MEMS resonator.

**Figure 12:**
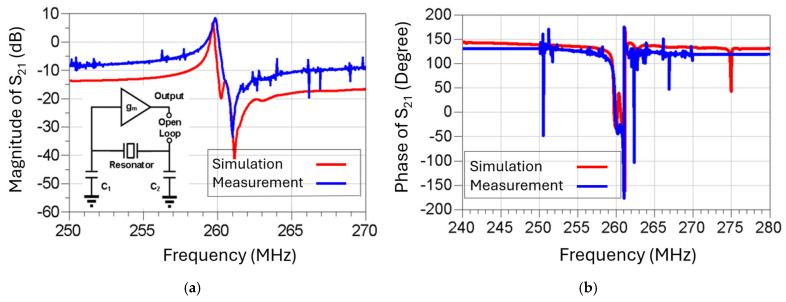
Open-loop frequency responses of the (**a**) magnitude and (**b**) phase of the serially cascaded MEMS resonator-sustaining amplifier circuit by ADS simulation and measurement.

In the simulation, we utilize the equivalent circuit model as shown in Figure 6b to represent the frequency response of the dual-mode resonator. Figure 12 shows that the simulated open-loop response (in red) matches fairly closely with the measured results (in blue) at 260 MHz in magnitude and phase, respectively. It is noted that the noise floor of the measured oscillation outputs is roughly 7–8 dB higher than the noise in the simulation, which is expected because some other noise sources are not considered in the simulation, such as thermal noise, transistor noise, or contributions from the RF probes and cables. In addition to that, the simulation result does not cover any spurious modes of the resonator since we apply the dual-mode equivalent circuit model depicted in Figure 6b.

**Figure 13:**
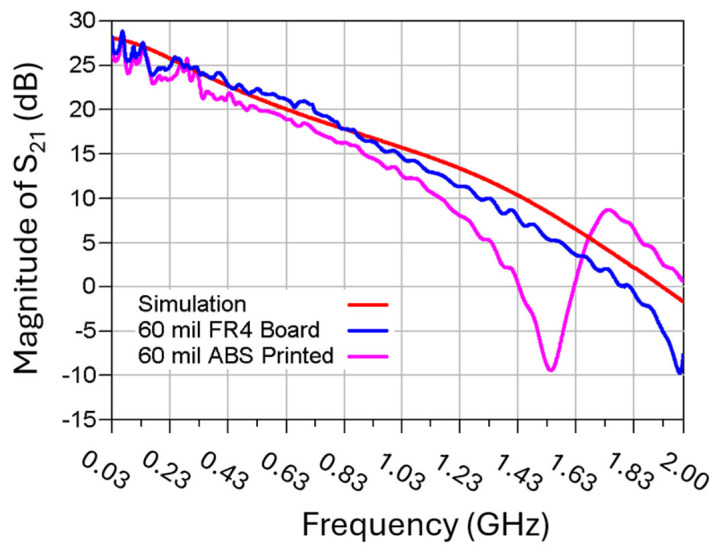
Comparison of the open-loop gain vs. frequency of the resonator-sustaining amplifier by ADS simulation and RF measurements for both FR4 PCB and 3D-printed ABS oscillator circuits.

Figure 13 presents the open-loop gain of the serially cascaded resonator-sustaining amplifier circuit by ADS simulation and measurement. As can be seen, the measured open-loop gain for a flame retardant (FR4) PCB board design is slightly higher than that of a 3D-printed ABS assembly. The power open-loop gain of the 3D-printed ABS assembly results in a lower oscillation output power of 0.6 dB.

**Figure 14:**
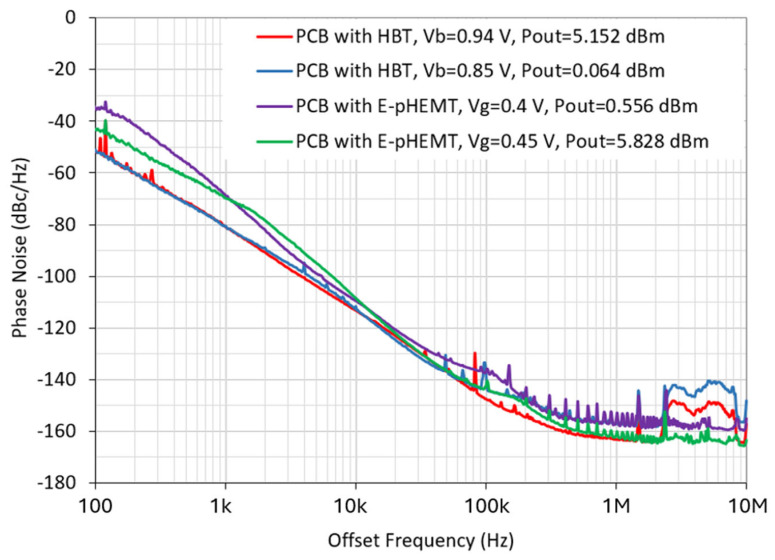
Comparison of measured phase noise performance of two oscillator designs at 260 MHz implemented on PCB boards with HBT and E-pHEMT transistors under different bias conditions.

The phase noise measurement is performed using a Keysight E5502A phase noise analyzer. Two types (CEL SiGe HBT and Avago E-pHEMT) of different commercial transistors are selected in this work due to their low flicker noise for the purpose of comparing their impacts on the oscillator performance. Both HBT and E-pHEMT transistors are integrated based on the Pierce oscillator circuit configuration over a PCB board to generate a measured oscillation frequency of 260 MHz. It is observed that the phase noise of the oscillator implemented using a SiGe HBT transistor is significantly better than that of the E-pHEMT counterpart at offset frequencies lower than 10 kHz due to the lower corner frequency of the HBT transistor, as shown in Figure 14. Meanwhile, the oscillator with an E-pHEMT biased at 0.45 V of gate voltage exhibits superior far-from-carrier phase noises among the four designs at offset frequencies above 1 MHz. Thus, an HBT transistor-based Pierce oscillator with high oscillation output power is strategically chosen for the subsequent study.

**Figure 15:**
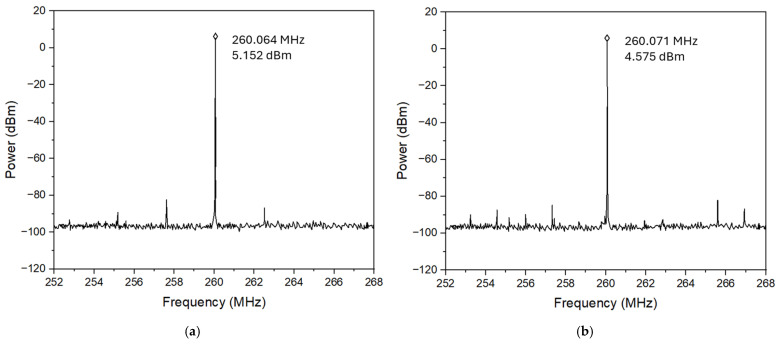
Comparison of the output amplitude measured for two 260 MHz oscillator design implementations: (**a**) PCB board with an HBT transistor with an output power of 5.152 dBm and (**b**) 3D-printed assembly with an HBT transistor with an output power of 4.575 dBm.

**Figure 16:**
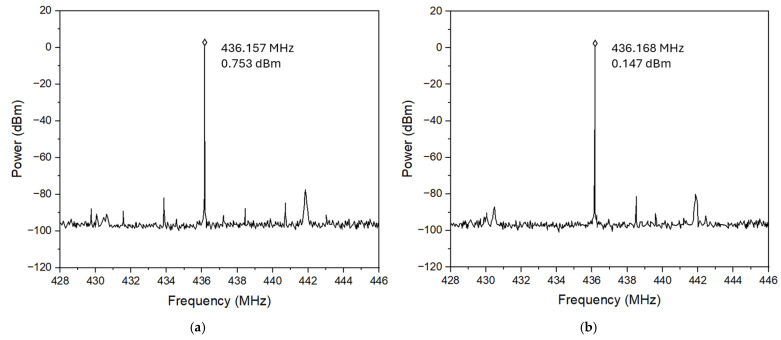
Comparison of the output amplitude measured for two 437 MHz oscillator design implementations: (**a**) PCB board with an HBT transistor with an output power of 0.753 dBm and (**b**) 3D-printed assembly with an HBT transistor with an output power of 0.147 dBm.

**Figure 17:**
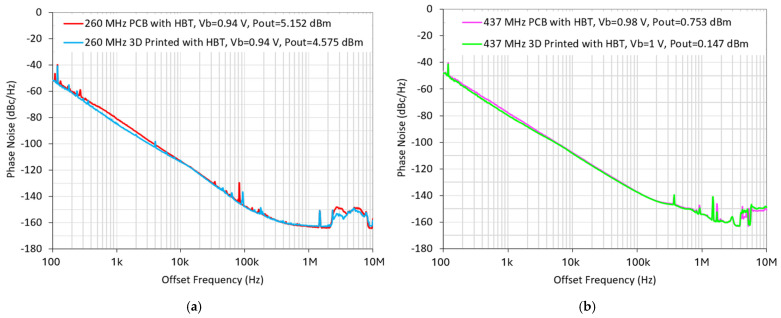
(**a**) The measured phase noise performance of two HBT-based 260 MHz Pierce oscillators implemented using PCB and 3D-printing technologies, and (**b**) the measured phase noise performance of two HBT-based 437 MHz Pierce oscillators implemented using PCB and 3D-printing technologies.

Figure 15 and Figure 16 show the measured phase noise responses of HBT-based Pierce oscillators operating at 260 MHz and 437 MHz, respectively, with oscillator circuits implemented by both traditional PCB technology and 3D-printed ABS chip-carrier. As shown, the Pierce oscillator implemented with an HBT transistor using PCB technology can achieve dual-frequency outputs at 260 MHz and 437 MHz, while producing the output powers of 5.152 dBm (as shown in Figure 15a) and 0.753 dBm (as shown in Figure 16a), respectively. Meanwhile, the oscillator integrated with an HBT transistor over a 3D-printed ABS substrate can be biased to generate oscillation outputs of 4.575 dBm at 260 MHz (as seen in Figure 15b) and 0.147 dBm at 437 MHz (as seen in Figure 16b). For comparison, all MEMS oscillator measurements were performed by employing the same dual-mode MEMS resonator as the tank circuit. It is worthwhile mentioning that the oscillator implemented by an HBT transistor, together with a 3D-printed ABS chip-carrier assembly at both 260 MHz and 437 MHz, showed slightly improved close-to-carrier phase noise performance, which is 2–3 dB lower than that of its PCB counterpart, as seen in Figure 17.

**Figure 18:**
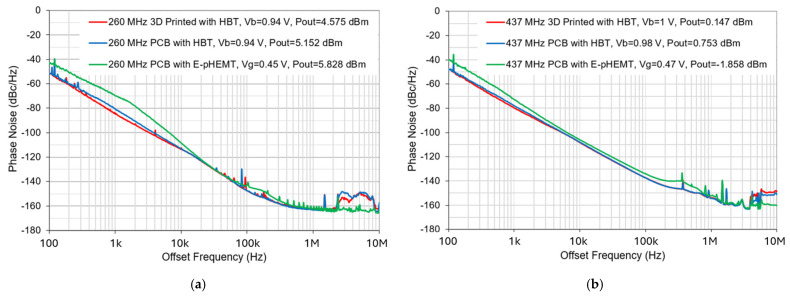
(**a**) Comparison of measured phase noise performance of three oscillator designs at oscillation frequency of 260 MHz, and (**b**) comparison of measured phase noise performance of three oscillator designs at oscillation frequency of 437 MHz.

By comparing the Pierce oscillator implemented with different transistors, as can be seen in Figure 18, we have demonstrated that designs with SiGe HBT exhibit significantly better phase noise performance than those of the E-pHEMT counterpart (in green). For oscillation output at 260 MHz and 437 MHz, a greater than 11 dB and 5 dB close-to-carrier phase noise reduction was observed for the oscillator design implemented with an HBT transistor compared to an E-pHEMT transistor design. Based on performance comparisons depicted in Figure 14 and Figure 18, Pierce oscillators implemented with an HBT transistor based on both traditional PCB technology and a 3D-printed ABS chip-carrier substrate result in overall better phase noise performance at frequency offsets up to 1 MHz. It is also observed that both close-to-carrier and far-from-carrier phase noise performance degrades at the MEMS oscillator output frequency of 437 MHz, which can be ascribed to relatively lower MEMS resonator quality factor and reduced oscillation amplitude compared to at 260 MHz.

Table 1 and Table 2 compare the measured phase noise performance of three types of Pierce oscillators implemented with both SiGe HBT and E-pHEMT transistors, together with either traditional PCB technology or 3D-printed ABS chip-carrier substrate. Table 1 shows that oscillators designed with HBT transistor have achieved 5–11 dB better phase noise than that of E-pHEMT transistor counterpart when implemented by traditional PCB technology. For instance, the oscillator with SiGe HBT transistor implemented on a PCB features a phase noise of −80.9 dBc/Hz at 1 kHz offset at 260 MHz and −77.8 dBc/Hz at 1kHz offset at 437 MHz, which are 11 dB and 5 dB better than those of E-pHEMT design. Moreover, HBT transistor-based Pierce oscillator implemented with 3D-printed ABS chip-carrier substrate has exhibited further performance enhancement of 2–3 dB beyond the measured phase noise response of a PCB implementation. Specifically, HBT transistor-based Pierce oscillator with 3D-printed ABS chip-carrier assembly has achieved phase noise of −84.2 dBc/Hz at 1 kHz offset and −79.9 dBc/Hz at 1 kHz offset when operating at 260 MHz and 437 MHz of output oscillation frequencies, as shown in Table 2.

This slightly reduced phase noise can probably be ascribed to the lower packaging- related losses of the 3D-printed ABS chip-carrier with a design strategy to mitigate the signal losses in the RF transmission lines over the operation frequency range. It is also noted that the oscillator with 3D-printed circuit implementation delivers an output power of 4.575 dBm at 260 MHz and 0.147 dBm at 437 MHz, which is only slightly lower than that of PCB counterpart. Given the strong correlation between the far-from-carrier phase noise floor level and oscillation amplitude, it is anticipated for all three oscillator designs with the same dual-mode MEMS resonator tank circuit and similar oscillation output amplitudes to exhibit comparable far-from-carrier phase noise of −163 dBc/Hz at 260 MHz and −154 dBc/Hz at 437 MHz, respectively.

The measured phase noise is largely on par with that reported in prior works, as shown in Table 3 [28,54,55,56,57,58,59,60,61,62]. It is worthwhile mentioning that this lateral-extensional mode resonator was designed to be able to operate with the dual modes while suppressing the other spurious modes, thanks to the adoption of thirteen side-supporting anchors at the nodal locations of one of the target acoustic resonance modes. But it is widely studied that each of the side-supporting anchors contribute with some levels of anchor- related energy dissipation, even when they are located at the nodal locations of one specific resonance mode. As a result, the MEMS resonator under spurious mode free operation does not exhibit highest *Q* that can be achieved by this MEMS device technology. Instead, we focus on suppressing other spurious modes so that the MEMS resonator tank circuit would only offer two resonance modes for the ease of oscillator design.

**Figure 19:**
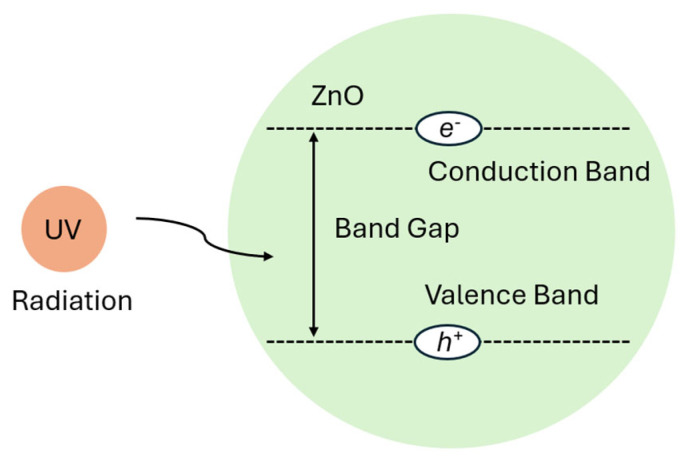
A schematic diagram to illustrate electron-hole transfer between the valence band and conduction band with a ZnO layer as a high bandgap semiconductor material, which can typically be excited by external energy sources such as UV radiation.

ZnO piezoelectric material plays a pivotal role in the operation of the piezoelectrically transduced MEMS resonators. Within the scope of this paper, the interaction between the frequency response of a piezoelectric resonator and the electron-hole pair generated within the ZnO as a high-bandgap semiconductor material is not investigated. However, it is worthwhile mentioning that we are aware of prior works that explore the effects of the ultraviolet (UV) or other light source radiation [63,64,65,66,67] on ZnO material to generate electron-hole pairs as a means of light-induced charge transfer, as seen in Figure 19.

## 4. Conclusions

A dual-mode MEMS resonator-based Pierce oscillator has been designed and implemented with both traditional printed-circuit-board (PCB) technology and 3D-printed acrylonitrile butadiene styrene (ABS) chip-carrier substrate. ZnO-on-SOI piezoelectrically transduced lateral-extensional mode MEMS resonator has been designed and microfabricated to generate two distinct, low insertion loss, high-*Q* resonance modes at 260 MHz and 437 MHz. By employing the dual-mode MEMS resonator as the tank circuit, three Pierce oscillator designs based on HBT and E-pHEMT transistors have been strategically designed and implemented by using PCB technology and 3D-printed ABS substrate. To facilitate oscillator circuit design and characterization, a complete dual-mode equivalent circuit model for the MEMS resonator has been extracted and experimentally validated by rigorously comparing measured frequency characteristics with simulated response by using Advanced Design System (ADS). Excellent agreement between model simulation and measurement has been achieved for time-domain oscillation output waveform as well as open-loop gain of serially cascaded MEMS resonator and sustaining amplifier circuit. The oscillator designs based on HBT transistor implemented with both PCB and 3D-printing technologies have demonstrated superior performance as compared to that of E-pHEMT counterpart with PCB implementation. Oscillator with HBT transistor implemented on a PCB features a phase noise of −80.9 dBc/Hz at 1 kHz offset at 260 MHz and −77.8 dBc/Hz at 1 kHz offset at 437 MHz, which are 11 dB and 5 dB better than those of E-pHEMT design. Furthermore, HBT transistor-based Pierce oscillator implemented with 3D-printed ABS chip-carrier substrate has achieved phase noise of −84.2 dBc/Hz at 1 kHz offset and −79.9 dBc/Hz at 1 kHz offset when operating at 260 MHz and 437 MHz of output oscillation frequencies, which corresponds to 2–3 dB phase noise enhancement beyond the measured response of a PCB implementation. All three MEMS Pierce oscillator designs exhibit comparable far-from-carrier phase noise of −163 dBc/Hz at 260 MHz and −154 dBc/Hz at 437 MHz, respectively. Overall, the MEMS Pierce oscillator implemented with HBT transistor circuits and 3D-printed ABS chip-carrier substrate implementation has achieved measured phase noise performance on par with those reported in prior works.

## Figures and Tables

**Table 1:** Phase noise comparison of HBT and E-pHEMT-based oscillators implemented by PCB.

	PCB with SiGe HBT		PCB with E-pHEMT	
Offset Frequency (Hz)	@ 260 MHz dBc/Hz	@ 437 MHz dBc/Hz	@ 260 MHz dBc/Hz	@ 437 MHz dBc/Hz
1 k	−80.9	−77.8	−69.8	−72.8
10 k	−113.1	−107.9	−108.4	−106.1
100 k	−147.4	−137.4	−142.7	−134.14
1 M	−163.1	−154.3	−161.4	−152.4

**Table 2:** Phase noise comparison of HBT oscillators implemented by PCB and 3D-printed ABS.

	PCB with SiGe HBT		3D-Printed ABS with SiGe HBT	
Offset Frequency (Hz)	@ 260 MHz dBc/Hz	@ 437 MHz dBc/Hz	@ 260 MHz dBc/Hz	@ 437 MHz dBc/Hz
1 k	−80.9	−77.8	−84.2	−79.9
10 k	−113.1	−107.9	−112.5	−108.3
100 k	−147.4	−137.4	−147.7	−137.4
1 M	−163.1	−154.3	−162.4	−154.2

**Table 3:** Overall performance comparison to prior works, including FOM and phase noise.

Reference	*f*_0_ (MHz)	*Q*	FOM (*f*_0_ × *Q*)	P_0_ (dBm)	Phase Noise (dBc/Hz)	Technology
This work	260 437	1171.3 689.2	0.305 × 10^12^ 0.301 × 10^12^	4.575 0.147	−84.2 @ 1 kHz and −162.4 @ 1 MHz −79.8 @ 1 kHz and −154.2 @ 1 MHz	Dual-Mode ZnO MEMS Pierce Oscillator with 3D-Printed ABS Chip-Carrier Substrate
[28]	176 222 307 482	1500 2100 1400 850	0.264 × 10^12^ 0.466 × 10^12^ 0.430 × 10^12^ 0.410 × 10^12^	−4.7 −4.8 −6.7 −13.6	−79 @ 1 kHz and −153 @ 1 MHz −88 @ 1 kHz and −160 @ 1 MHz −84 @ 1 kHz and −151 @ 1 MHz −68 @ 1 kHz and −145 @ 1 MHz	AlN Contour-Mode MEMS Oscillator AMIS 0.5 μm 5 V CMOS
[54]	472 1900	1800 600	0.850 × 10^12^ 1.140 × 10^12^	−6.5 −16	−82 @ 1 kHz and −160 @ 1 MHz −69 @ 1 kHz and −153 @ 1 MHz	Dual-Mode AlN MEMS 0.5-µm CMOS
[55]	1500	1573	2.360 × 10^12^	−14	−86 @ 1 kHz and −147 @ 1 MHz	Temperature-Compensated FBAR 0.35 µm CMOS
[56]	150	10000	1.500 × 10^12^	−9	−96 @ 1 kHz and −128 @ 1 MHz	Disk MEMS Oscillator Encapsulated with AGC Module
[57]	5.996	590	0.004 × 10^12^	−25	−80 @ 1 kHz and −115 @ 100 kHz	FC and CC Beam Capacitive Resonator 0.18 µm CMOS
[58]	3.25	115	0.004 × 10^11^	24	−115 @ 1 kHz and −145 @ 1 MHz	Flexural-Mode MEMS Two-Port Resonator Array
[59]	283	1100	0.311 × 10^12^	N/A	−102.6 @ 1 kHz and −164.6 @ 1 MHz	MEMS Lamb Wave Resonator on X-cut Lithium Tantalate
[60]	17.55	5000	0.088 × 10^12^	−3.64	−137 @ 1 kHz and −158 @ 1 MHz	AlN-Si WE TSMC 0.18 μm CMOS
[61]	16.75	5550	0.093 × 10^12^	N/A	−124.4 @ 1 kHz and −160.4 @ 1 MHz	AlN WE-Mode MEMS Based on STT
[62]	10	491580	4.916 × 10^12^	−6	−156 @ 1 kHz and −161 @ 1 MHz	SC-cut Crystal Oscillator with Transformer-Coupled Differential Amplifier

## Data Availability

Measurements and other relevant data are available from the corresponding authors upon request via email.
